# Internet use, users, and cognition: on the cognitive relationships between Internet-based technology and Internet users

**DOI:** 10.1186/s40359-023-01041-5

**Published:** 2023-03-27

**Authors:** Vishruth M. Nagam

**Affiliations:** Stony Brook University, Stony Brook, USA

**Keywords:** Transactive memory, Semantic memory, Internet, Recall

## Abstract

**Background:**

This study aims to investigate growing Internet use in relation to memory and cognition. Though literature reveals human capability to utilize the Internet as a transactive memory source, the formational mechanisms of such transactive memory systems are not extensively explored. The Internet’s comparative effects on transactive memory and semantic memory are also relatively unknown.

**Methods:**

This study comprises two experimental memory task survey phases utilizing null hypothesis and standard error tests to assess significance of results.

**Results:**

When information is expected to be saved and accessible, recall rates are lower, regardless of explicit instructions to remember (Phase 1, N = 20). Phase 2 suggests the importance of order of attempted recall: depending on whether users first attempt to recall (1) desired information or (2) the information’s location, subsequent successful cognitive retrieval is more likely to occur for (1) only desired information or both desired information and location thereof or (2) only desired information’s location, respectively (N = 22).

**Conclusions:**

This study yields several theoretical advances in memory research. The notion of information being saved online and accessible in the future negatively affects semantic memory. Phase 2 reveals an adaptive dynamic—(1) as Internet users often have a vague idea of desired information before searching for it on the Internet, first accessing semantic memory serves as an aid for subsequent transactive memory use and (2) if transactive memory access is successful, the need to retrieve desired information from semantic memory is inherently eliminated. By repeatedly defaulting to first accessing semantic memory and then transactive memory or to accessing transactive memory only, Internet users may form and reinforce transactive memory systems with the Internet, or may refrain from enhancing and decrease reliance on transactive memory systems by repeatedly defaulting to access only semantic memory; the formation and permanence of transactive memory systems are subject to users’ will. Future research spans the domains of psychology and philosophy.

**Supplementary Information:**

The online version contains supplementary material available at 10.1186/s40359-023-01041-5.

## Background

The Internet is a relatively recent invention, having been established in 1983, yet its reach has extended across the globe [1]. Over the past 2 decades, the world has seen a distinct increase in the number of people connected to the Internet (from 5 to 58.7%) [2]. Furthermore, due to the COVID-19 pandemic, many in-person activities have been shifted to virtual Internet-based mediums. The Internet has become a part of daily life for many individuals, and thus it is necessary to investigate the Internet’s potential cognitive implications.

“I don’t know; ‘Google’ it!” has become a common household remark in response to a question that cannot be answered at the moment. With the Internet being accessible through various means of technology, people often utilize search engines with complex algorithms to find any needed information. Regardless of whether people have to recall the precise quantities of ingredients in a recipe or how many points a basketball player scored in a game, they instantly search for it on the Internet using smart devices. People may experience withdrawal symptoms when they cannot instantly gratify themselves with the information they need, and thus some have even been led to believe that modern students, surrounded by various ways to access the Internet, are declining in intelligence and memory [3].

Throughout history and civilization, humans have relied on each other to share the burden of a cognitive task (e.g. remembering complex information), which has resulted in specialization in society and within relationships. Transactive memory refers to the idea that people develop a “system of encoding, storage, and retrieval of information from different knowledge domains.” This type of memory includes both the source of the information along with knowing the process of how to access or ask for that information when it is needed [4–7]. Thus, the “transactions” among individuals, as well as among the knowledge bases themselves, make up a transactive memory system.

In contrast, semantic memory is the long-term declarative memory of general facts and data. It consists of “cultural knowledge, ideas, and concepts” that have been accumulated throughout one’s lifetime [4, 8]. Some examples of information falling under semantic memory may include the names of the most populous cities, the historical significance of a certain war, or basic multiplication and division rules. Though one may be able to store the same kind of information in working memory (i.e. short-term memory), the oft-ignored difference between working memory and semantic memory is that the latter allows for the long-term storage (more than a few seconds) of the information, which involves not only the hippocampus but also a vast network of cortical regions [4].

In previous studies, it has been determined that humans are capable of forming transactive memory systems, or collective storages of information outside of themselves, with the Internet [3, 7]. However, the Internet’s effects on transactive memory in comparison to semantic memory have not been extensively explored; in addition, the mechanisms of how transactive memory systems with the Internet are established and strengthened are relatively unknown (see “Hypothesis” Section for further elaboration). This study investigates how the Internet plays a role in a transactive memory system with Internet users of the modern generation, the Internet’s comparative effects on transactive memory and semantic memory, and whether the cognitive implications of the aforementioned effects can help explain how transactive memory systems are formed and established. It was around these central aims that the hypothesis was framed.

### Hypothesis

The author’s hypothesis about human cognitive relationships with the Internet can be summarized in four parts to be tested in two experimental phases (Phases 1 and 2): (1) if it is expected that information will be saved and accessible in the future, people are less likely to recall the information (tested in Phase 1), (2) if it is expected that information will be saved in a known location, the memory of the location where the information is saved (transactive memory) is more enhanced than the memory of the information itself (semantic memory), as the location (e.g. a website name or a short folder name) may be more memorable (tested in Phase 2), (3) explicit instructions to remember do not have a significant effect on memory and recall (tested in Phase 1), and 4) if it is expected that information will be saved in a known location, the order of attempted recall may not have a significant effect on memory, if the memory questions are worded such that attempting to recall information does not influence attempts to recall the information’s location and vice versa (tested in Phase 2).

Hypothesis Parts 1, 2, and 3 are based on and serve to validate the findings of previous transactive memory research and theory and of the Internet’s known comparative effects on transactive memory and semantic memory [7, 9]. In particular, Sparrow et al. show that transactive memory is enhanced in comparison to semantic memory, but only test access of transactive memory following prior access of semantic memory [9]. Thus, as an unexplored point of experimentation, Hypothesis Part 4 (testing order of attempted recall) was included in this study to potentially yield greater insight into the comparative effects of Internet use on transactive memory and semantic memory and into the formational mechanisms of transactive memory systems. Hypothesis Part 4 assumes the null hypothesis, as a sufficient base of evidence suggesting otherwise was not found.

## Materials and methods

Twenty and twenty-two human volunteers participated in Phase 1 and Phase 2, respectively. All participants had a means of digitally accessing Google Forms (via Internet-connected devices). A stopwatch was used to ensure participants completed each step of the experiment in the allotted times. A calculator and spreadsheets software were used to perform data analysis.

Twenty-two students enrolled in Folsom Cordova Unified School District middle schools were administered the experimental memory task surveys. Parents and/or legal guardians of the students completed the Human Informed Consent Form, which contained brief information on the study. Phase 1 data was not available for two students.

### Phase 1

All the participants first read thirty trivia-style statements on a Google Form (refer to Additional file 1: Appendix B.1 for a digital copy). The participants were divided into two groups: half were told to remember the statements while the other half was not given any explicit memory instructions, in order to simulate attempting to recall information found on the Internet with and without anticipating that the information will be needed and/or tested in the future, respectively (refer to Additional file 1:Appendix F for a participant flow diagram depicting how the hypothesis was addressed in the steps of Phase 1) [3, 7].

Half of the statements were labeled as “Will Be Saved” and the other half as “Will Be Erased” on the Google Form. A ten-minute reading period was given for the participants to memorize the statements. By mentioning that only the “Will Be Saved” statements would be accessible later, the perception was created that the “Will Be Saved” statements would be available for future reference and that the “Will Be Erased” statements would not be accessible after the reading period (see Additional file 1: Appendix B.3).

After the reading period, the participants were tested on their memory of the statements in an uncued recall format, which was chosen to prevent wording bias. The participants had ten minutes to type as many statements as they could remember into the Google Form (see Additional file 1: Appendix B.2). Such quantification of memory of both groups of participants allows for an assessment of the first two parts of the hypothesis [7]. For example, to quantify the memory of saved statements (statements labeled as “Will Be Saved”) for the participants who were given explicit memory instructions, the calculation “(number of saved statements remembered by the participants with explicit memory instructions)/(total number of saved statements)” would be performed. The control in this experiment is the memory of the saved statements for the participants without any explicit memory instructions, as normally when using the Internet, people do not make conscious efforts to remember, and they know that the information they view is saved online, for instance, in a web page.

The purpose of explicitly telling only half of the participants to remember the statements and refraining from any memory instruction for the other half of the participants is to simulate attempts to remember online information when expecting or not expecting, respectively, the information will be needed and/or tested in the future. Thus, Phase 1 determines how the expectation of information being saved online and accessible in the future via Internet-based technology, as well as how being explicitly asked to remember, may influence semantic memory.

### Phase 2

Phase 2 sought to determine if, with the expectation that the information will be saved in a known location, participants are more likely to remember where the information can be found (transactive memory) rather than the information itself (semantic memory). Phase 2 also investigates if the order of attempted recall (attempting to recall the information before its location, or vice versa) would have a significant effect on the participants’ memory (refer to Additional file 1: Appendix F for a participant flow diagram depicting how the hypothesis was addressed in the steps of Phase 2).

Participants first read a list of thirty trivia-style statements (different from the list used in Phase 1) in random order on a Google Form (see Additional file 1: Appendix D.1 for a digital copy). The statements were already randomly saved to one of four folders, all of which were similarly named (“Information,” “Facts,” “Points,” “Figures”), or saved in no specific folder (the phrases “generically saved” and “saved in no specific folder” will be used interchangeably in the rest of this paper). The trivia-style statements will be randomly distributed across the total five information storage locations, and each storage location would thus have six—an equal number of—statements. The purpose of generically saving a portion of the statements was to eliminate the effects of the added memory toll of having to remember a statement and its folder location [4, 7]. A screenshot of all the folder locations in which these statements would be saved was given in the Google Form to ensure that the participants gained the perception that four-fifths of the statements are saved in their assigned folders and one-fifth of the statements are generically saved.

After a ten-minute reading period, participants were given another ten minutes to answer questions about all the statements and their folder locations phrased in a cued recall format on a Google Form (refer to Additional file 1: Appendix D.2). The cued recall format was used to simulate Internet use, as, when people try to recall information, or the location thereof, that they know is saved on the Internet, they generally tend to have at least a vague idea of the information at hand [5, 7]. In addition, the cued recall format for both semantic memory and transactive memory more accurately reflects real-world Internet use compared to previous Internet cognition studies, which only explore uncued recall with semantic memory and cued recall for transactive memory and thus exhibit implicit bias towards transactive memory [9]. Due to time constraints, the participants were tested on their memory of ten randomly selected statements and their folder locations (see Additional file 1: Appendix D.3), yielding a total of twenty questions (ten statement questions and ten folder location questions).

The questions about the exact statements were free-response (answers with slightly different wordings that still convey the same meaning were accepted). For example, if the statement “A bolt of lightning contains enough energy to toast 100,000 slices of bread” was saved in the “Points” folder, a question about the statement might be structured like the following: “Enter the statement about lightning to the best of your ability.” The average proportion of statements remembered by each participant was calculated with the following expression: “(average number of correct statements / total number of statements tested)”. A question about a statement’s folder location might be structured like the following: “In which folder was the statement about lightning saved?” Each folder location question would require a short answer, such as “Figures” or “No specific folder.” In the case of the lightning question, the participant would have to type “Points” to correctly answer the question. The average proportion of folder locations remembered by each participant was calculated with the following expression: “(average number of correct folder locations / total number of folder location questions tested)”.

However, the average proportions of remembered statements and folder locations can be somewhat misleading, as a participant may have a higher chance of recalling the folder location of a statement due to the previous recalling of the statement or vice versa. To investigate further, on the Google Form, half of the folder location questions preceded the exact statement questions, and the other half of the folder location questions followed the exact statement questions. For example, for the lightning statement above, the folder location question follows the exact statement question, but for another statement, the folder location question may precede the exact statement question. This would help determine if order matters in recalling the statement and its folder location, simulating how the order of one’s attempted recall of information’s online location versus the information itself may influence transactive and/or semantic memory.

In addition, with just the average proportions of remembered statements and folder locations, an accurate conclusion cannot be made about the memory of a specific piece of information, as a participant may remember the statement but not its location or vice versa. To compare the participants’ memories of each statement and its folder location, the author calculated the proportions of statements for which the participants recalled (1) neither the statement nor folder location, (2) the folder location but not the statement, (3) the statement but not the folder location, and (4) both the statement and its folder location. These four cases were analyzed for both the group of statements that had the exact statement questions given first and the group of statements that had the folder location questions given first, resulting in eight total statistical cases. For example, to calculate the participants’ memory of case 2 statements (only folder location of those statements were remembered) that had the preceding folder location question, the expression “(number of case 2 statements with the preceding folder location question/total number of statements with the preceding folder location question)” was used. The control in this experiment would be the case 1 statements with the preceding statement question, as usually people tend to attempt recalling the saved information first before resorting to searching it on the Internet and because the memory of the case 1 statements would serve as a comparison to the statements in case 2, 3, and 4.

In this way, Phase 2 will help to conclude how the expectation that the desired information’s digital location affects memory of the information (semantic memory) in comparison to memory of the information’s location (transactive memory). Phase 2 will also help to determine if attempting to recall a statement or its folder location first affects the recall of the other.

### Statistical analysis

Null hypothesis statistical tests were used to assess the statistical significance of the results of both Phases 1 and 2 to 95% confidence. One-tailed and two-tailed inferential t-tests were used depending on the nature of the hypothesis tested (e.g., as Hypothesis Part 1 poses significantly *less* recall when information is known to be saved, assessment of results pertaining to Hypothesis Part 1 warrants one-tailed statistical analyses. Assessments of Hypothesis Part 4 as the null hypothesis warrant two-tailed statistical analyses).

## Results

### Phase 1

**Fig. 1:**
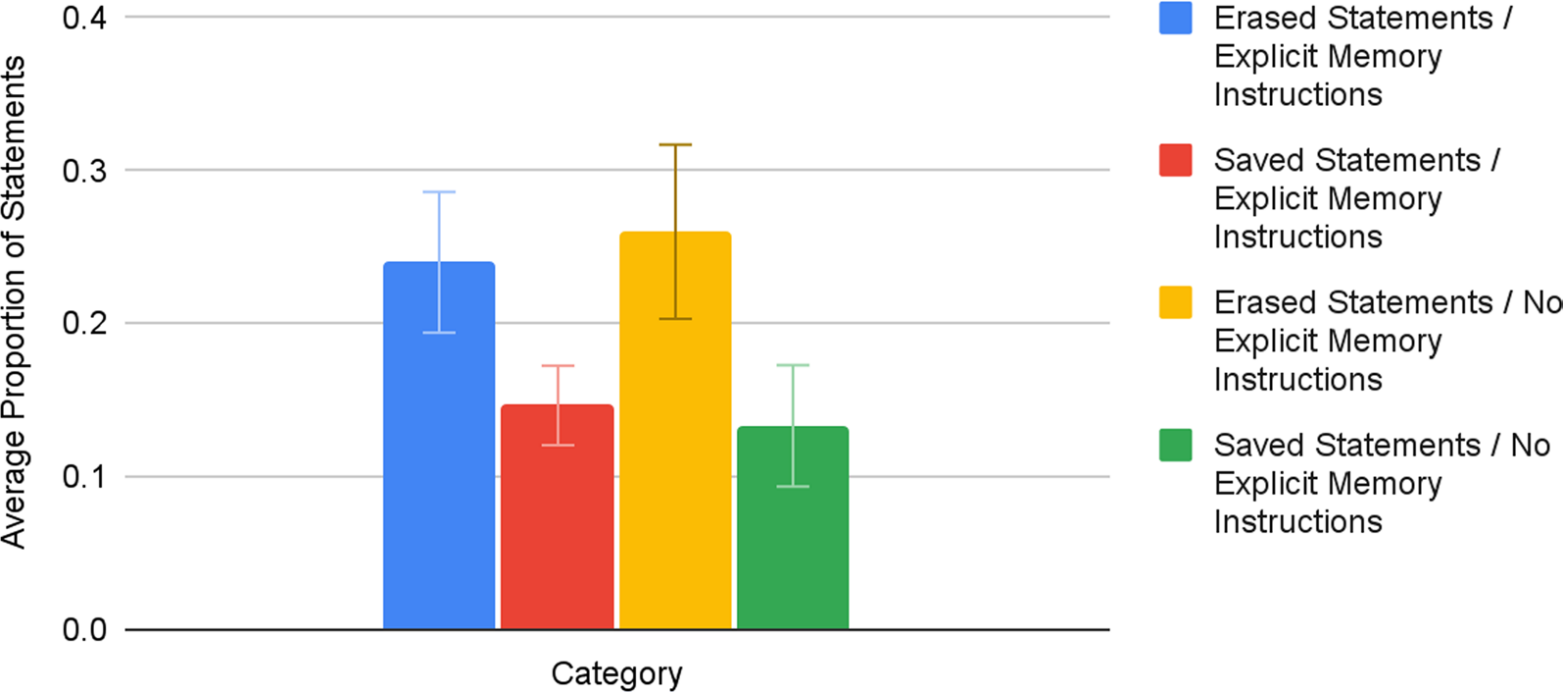
Proportion of “Will be Erased” and “Will be Saved” statements recalled, by the presence of explicit memory instructions.“Will be Erased” and “Will be Saved” statements are abbreviated as “Erased” and “Saved,” respectively. Error bars represent ± 1 SE_X_

The analysis of the results of Phase 1 (refer to Fig. 1; see Additional file 1: Appendix C for raw data), with Saved and Erased statement groups as well as explicit memory instructions and no memory instructions groups, showed that participants with explicit memory instructions (EMS) remembered the statements they believed to be erased (Erased/EMS M = 0.24, SE = 0.046) significantly better (t(14.235) = 1.775, *p* < 0.05, one-tailed unpooled t-test) than the statements they believed to be saved (Saved/EMS M = 0.147, SE = 0.026). Participants with no explicit memory instructions (NEMS) also remembered the Erased statements (Erased/NEMS M = 0.26, SE = 0.057) significantly better (t(16.016) = 1.81, *p* < 0.05, one-tailed unpooled t-test) than the Saved statements (Saved/NEMS M = 0.133, SE = 0.040). There were no significant differences in the memory of participants who received EMS and those who did not.

### Phase 2

**Fig. 2:**
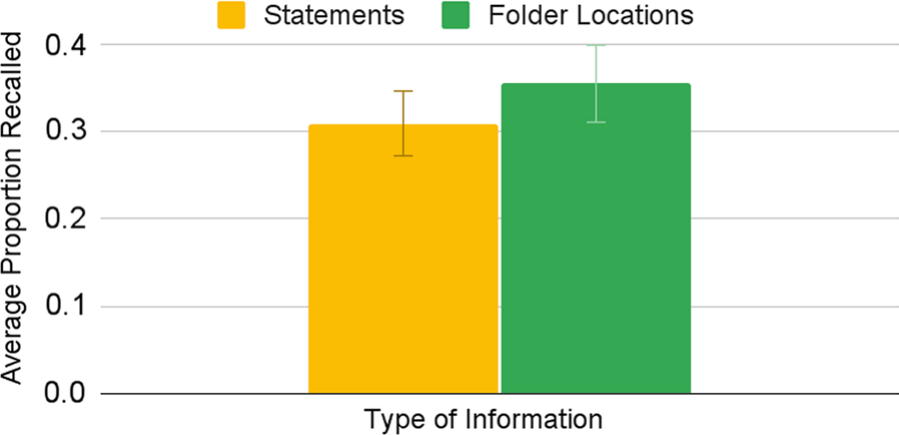
Overall proportions of statements and folder locations recalled. Error bars represent ± 1 SE_X_

**Fig. 3:**
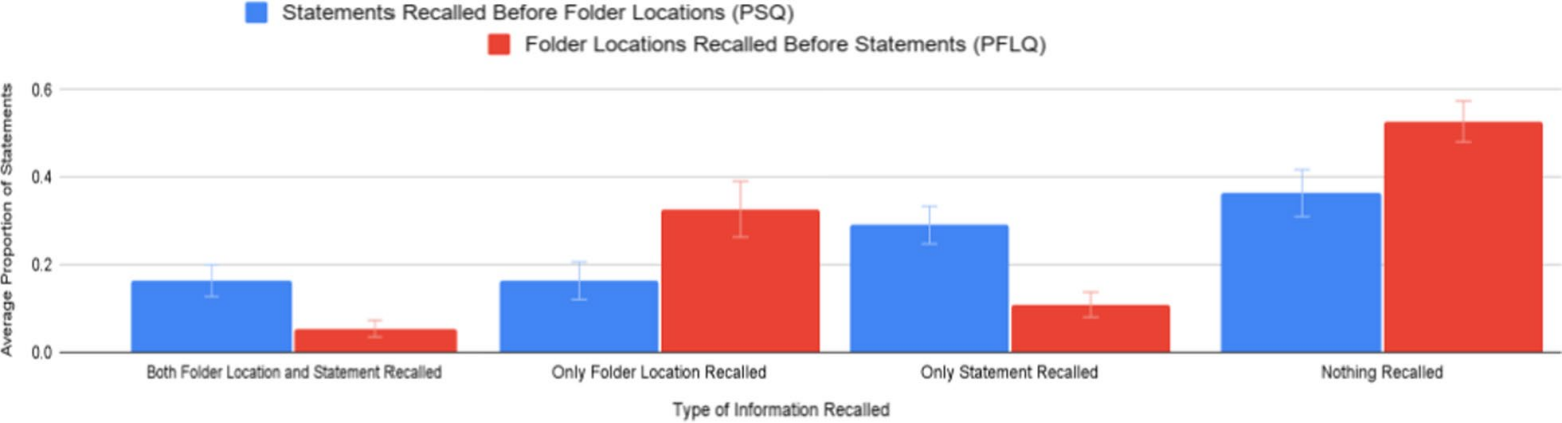
Proportion of statements and folder locations recalled, by order of recall and type of information recalled. Error bars represent ± 1 SE_X_

In Phase 2 (refer to Fig. 2), the participants correctly recalled on average 30.9% of the statements (SE = 0.037) and 35.4% of the folder locations (SE = 0.044); the difference between these values not being significant. Due to aforementioned reasons pertaining to the procedural steps of Phase 2 (refer to Procedural Steps for elaboration), the proportions of each of the four cases for the statements, both with the preceding statement question (PSQ) and the preceding folder location question (PFLQ), were calculated as well (refer to Fig. 3; see Additional file 1: Appendix E for raw data).

Participants were able to correctly recall both the statement and folder location (Both/PSQ M = 0.164, SE = 0.036) or only the folder location, but not the statement (Folder/PSQ M = 0.164, SE = 0.043) for relatively few statements with the PSQ. Participants were more likely to be able to recall only the statement, and not the folder location, (Statement/PSQ M = 0.291, SE = 0.043) or nothing at all (Nothing/PSQ M = 0.364, SE = 0.054) about the statements with the PSQ. The difference between the participants recalling only the statement and recalling only the folder location for the statements with the PSQ was significant (t(41.999) = − 2.090, *p* < 0.05, one-tailed unpooled t-test).

Participants were seldom able to recall both the statement and the folder location (Both/PFLQ M = 0.055, SE = 0.019) or only the statement, but not the folder location (Statement/PFLQ M = 0.109, SE = 0.029) for statements with the PFLQ. Participants were relatively more likely to be able to recall only the folder location, not the statement (Folder/PFLQ M = 0.327, SE = 0.064) or nothing at all (Nothing/PFLQ M = 0.527, SE = 0.047). The difference between the participants recalling only the folder location and recalling only the statement was significant (t(29.109) = − 3.121, *p* < 0.05, one-tailed unpooled t-test).

A comparison between the participants’ memory of the information about the statements with the PSQ and PFLQ is necessary to assess the fourth part of the hypothesis. Participants were significantly more likely to recall both the statement and the folder location (t(32.052) = 2.650, *p* < 0.05, two-tailed unpooled t-test), as well as only the statement and not the folder location (t(36.484) = 3.522, *p* < 0.05, two-tailed unpooled t-test), for statements with the PSQ than for the statements with the PFLQ. For statements with the PFLQ compared to statements with the PSQ, participants were significantly more likely to recall only the folder location, but not the statement (t(36.761) = -2.122, *p* < 0.05, two-tailed unpooled t-test), as well as nothing at all (t(41.199) = − 2.290, *p* < 0.05, two-tailed unpooled t-test).

## Discussion

The results from Phase 1 show that trivia statements believed to be erased were recalled significantly more than statements believed to be saved, regardless of the presence of explicit instructions to remember. People will not recall information they believe to be available to refer to later at the same rate as information believed to be erased; this may be due to the notion that they can look up any desired information using a search engine, thus eliminating the need to remember that piece of information. This result is similar to findings in directed forgetting studies, which have shown that people do not remember information they are told that they can forget as accurately as when they do expect the need to remember the information in the future [7, 9–11]. Explicit memory instructions did not significantly influence memory; thus, it is reflected that the expectation of information being saved and later accessible affects recall rates more than the anticipation that the information will be needed and/or tested in the future. This finding may correspond to those in previous studies regarding comparisons between incidental and intentional learning of information, which have generally reported that explicit instructions to remember do not significantly influence memory of information [7, 9, 12]. Phase 1 thus supports Hypothesis Parts 1 and 3.

In Phase 2, there was not a significant difference between the overall recall rates of statements and folder locations. However, the analysis of the four cases of statements reveals that participants were more likely to recall both the statement and its folder location or only the statement, if the statement question preceded the folder location question. Conversely, if the folder location question preceded the statement question, participants were more likely to recall only the folder location or nothing at all. This novel finding reflects the dependence of people’s ability to recall information based on the order of attempted recall. Essentially, when people attempt to recall the “what” first, they are more likely to remember both the “what” and the “where” or only the “what;” when people attempt to recall the “where” first, they are more likely to remember only the “where” or nothing at all. Thus, the results of Phase 2 uphold Hypothesis Part 2 in certain cases (when transactive memory is accessed first), and disprove Hypothesis Part 4.

## Conclusion

This study suggests that Internet-based technology may serve as a transactive memory source for the user, similar to how one could ask friends or colleagues to obtain any desired information. Semantic memory may be negatively impacted by the expectation that information will be saved and available for future reference, regardless of whether or not it is anticipated that the information will be needed or tested in the future (Phase 1). This study makes the novel proposition that the order of attempted recall (first attempting to recall the desired information versus its Internet location) affects Internet users’ rates of recall (Phase 2).

As an increasing proportion of our society plugs into the Internet, more and more users are forming interconnected transactive memory systems, not with each other, but with the Internet through various means of technology. Similar to how people remember to ask a friend in case they forget a homework assignment or to reach out to a colleague for the latest updates on a project, people are remembering the sorts of information the Internet holds and how to access it through our devices, rather than the information itself. Phases 1 and 2 together suggest that the common perception of the declining memory of society as a whole may be invalid, as we may simply be more frequently exercising a new type of memory—transactive memory rather than semantic memory [8]. Internet users are remembering more of how to navigate the Internet and focus on what they need to find, which may prove to be a useful skill in this age of modernization, when people are often bombarded with a constant influx of information from various online sources.

Phase 2 suggests, when transactive memory is accessed first, subsequent successful retrieval of information is more likely to occur from only transactive memory or not at all. A possible explanation for this phenomenon may lie in proactive interference, by which the activation of the memory system accessed first (transactive memory) disrupts the subsequent activation of and recall of information from another memory system (semantic memory), or an adaptive use of memory (see next paragraph for further elaboration). In addition, Phase 2 suggests that, when semantic memory is first accessed, subsequent successful retrieval of information may be enhanced for only semantic memory or both semantic memory and transactive memory. Thus, by repeatedly defaulting to first access semantic memory then transactive memory or first access only transactive memory, Internet users may build and strengthen transactive memory systems with the Internet—or, by defaulting to semantic memory without subsequently attempting to access transactive memory such that transactive memory is not activated, may refrain from enhancing and decrease reliance on transactive memory systems. This study proposes the novel observation that transactive memory systems with the Internet may be willingly formed and established, but not permanent (see “Limitations and Future Directions” Section).

It is also important to specifically note that when semantic memory is activated first, both semantic memory and transactive memory may be enhanced; however, if transactive memory is activated first, semantic memory is not enhanced. This novel finding may reflect an adaptive use of memory. As Internet users tend to have a vague idea of the online information desired before searching for it on the Internet, first attempting to recall the information itself from semantic memory may serve as an aid for subsequently recalling the information’s storage location from transactive memory (refer to Procedural Steps for a similar explanation of why cued recall was used). Conversely, if transactive memory and a transactive memory source are successfully first accessed, the need to access semantic memory for desired information is eliminated, as the desired information is now provided by the transactive memory source.

### Limitations and future directions

Limitations are part of the experimental scientific process. As participants were garnered on a voluntary basis (see Additional file 1: Appendix A for a digital copy of the Human Informed Consent Form), sampling bias may exist due to the possibility of the participants having stronger or weaker memory capacities than those of the majority of the human population of similar backgrounds. The sample size is not considered to be a limitation as the statistical analyses yielded significant findings assessing the fourfold hypothesis and leading to robust conclusions. Further research could study varying participant demographics, investigating how participant backgrounds may impact the formation and/or function of transactive memory systems.

In Phase 1, the participants were randomly split into two groups, with only one group receiving explicit memory instructions. There is a possibility that each of the groups as a whole may not have had similar memory capacities, which might have influenced the conclusion of the statistically insignificant effects of explicit memory instructions. Also, due to the memorable trivia-style nature of the statements in both Phases 1 and 2 (refer to Additional file 1: Appendices B.1 and D.3, respectively, for all of the statements in both phases), participants might have been able to recall the statements at higher rates than if the statements were less memorable. This can be explored in future studies by having participants read multiple lists of relatively ordinary sentences and testing them on their memory of the sentences and where the sentences can be found.

The results of Phase 2 present significant potential and interest for further research. For example, studies may investigate the degree of permanence of transactive memory systems. From the perspective of cognitive neuroscience, studies could also investigate the neural changes that may potentially contribute to the strengthening or weakening of transactive memory systems. The effect of “relatedness” between desired information and corresponding Internet storage locations could also be explored in future research.

### Implications

The philosophical bases for how Internet use is to be understood has been called into question. Yet, notwithstanding the numerous perspectives—including of transactive memory, extended memory, and memory scaffolds—that have been brought into such discussion, this study furthers the understanding of psychological phenomena at play in digital, Internet-based knowledge acquisition [8, 13–15]. The presented findings would still hold regardless of which perspective is accepted.

The choice to administer experimental memory task surveys via Google Forms reflects the fundamental aims of the study design—to simulate Internet use through the “online” nature of the administered surveys, the use of “Google” services, and a completely digitized study design able to be completed on any Internet-connected device reflecting the Internet’s decentralization. Although transactive memory systems may take varying forms even within Internet use (e.g., cognitive associations with hyperlinks and site maps), the fundamental nature of Internet use in a transactive memory system—associating certain “keywords”, locations, hyperlinks, or website names (rather than URLs) that store and/or which lead to the desired information—is consistent, suggesting applicability of this study’s findings to digital, Internet-based means of knowledge and information exchange [16, 17]. Yet, studies have indicated differences in how and when such Internet features effectively improve (semantic) memory of desired information [18]. This study’s design may be akin to what has been termed by some authors as “site maps” or “knowledge maps” (see Additional file 1: Appendices B and D for visual tables of statements provided in memory task surveys), providing users a holistic, often visual representation of the Internet information landscape of interest before memory is assessed. Thus the implications of this study’s findings may be relevant for at least such site maps.

This study sheds light on the ethics of psychological research and gives rise to relevant questions for consideration. Ethical and philosophical topics, issues, and dilemmas relevant to the novel findings of this study include but are not limited to: mental health and declining social interaction with human transactive memory sources (friends, colleagues, etc.), causality and impact analysis of disparities in Internet accessibility, impact of Internet-based transactive memory use on sense of self and relevant perspectives (e.g., extended mind perspective), privacy and informed consent in memory modification, cognitive responsibilities (e.g., to remember or forget) in social settings given the default of Internet-based devices to store or “technologically remember” information, permanence of transactive memory systems with the Internet, and humanity in an age of rapid technological progress [4, 8, 19–21].

Such concerns involve careful scientific, ethical, legal, and social judgement [4]. Considering topics such as those discussed above will be beneficial for the sectors of science, government, and the public to establish strong and agreeable ethical boundaries and ensure equity and social justice as society progresses into the future.

## Supplementary Information

**Additional file 1**. Appendices

## Data Availability

All data generated during this study are included in this article and its supplementary information files.
