# Supplementary material for: Internet use, users, and cognition: on the cognitive relationships between Internet-based technology and Internet users
Source: BMC Psychol. 2023 Mar 27;11:82. doi: 10.1186/s40359-023-01041-5 (PMC10042401; doi:10.1186/s40359-023-01041-5)
Supplement: Supplementary file 1 — Additional file 1. Appendices [file 40359_2023_1041_MOESM1_ESM.pdf]

Appendices

Appendix A

Human Informed Consent Form

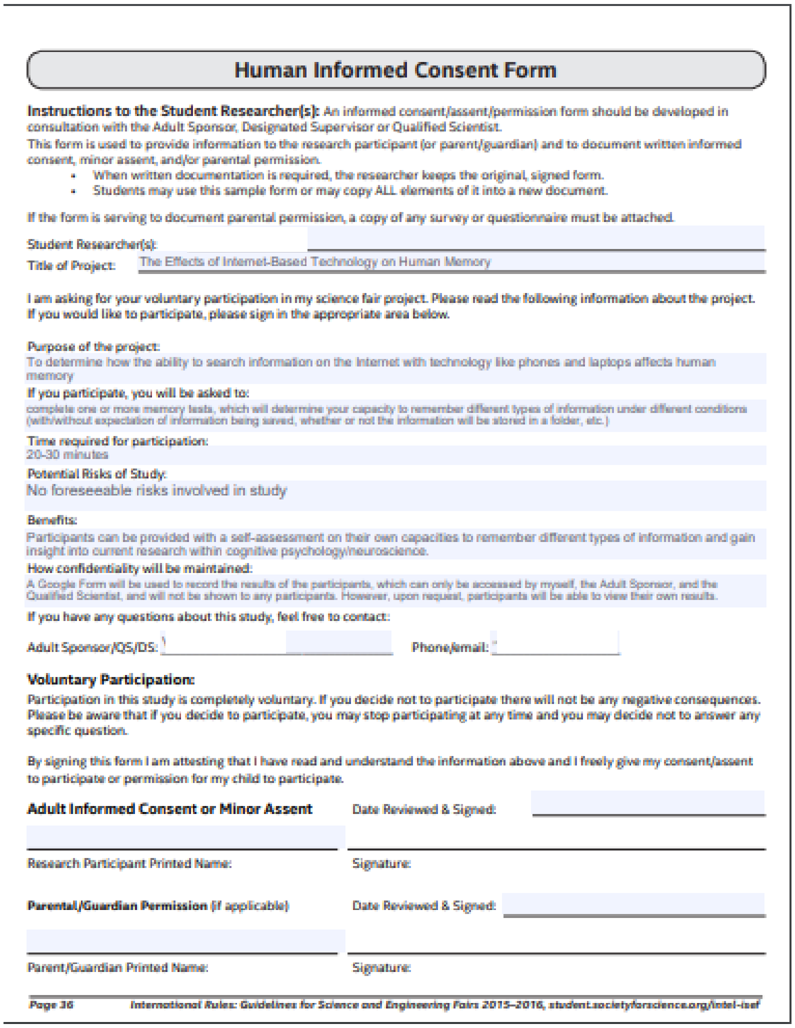

Human Informed Consent Form

Instructions to the Student Researcher(s): An informed consent/assent/permission form should be developed in consultation with the Adult Sponsor, Designated Supervisor or Qualified Scientist.
This form is used to provide information to the research participant (or parent/guardian) and to document written informed consent, minor assent, and/or parental permission.

- When written documentation is required, the researcher keeps the original, signed form.
- Students may use this sample form or may copy ALL elements of it into a new document.

If the form is serving to document parental permission, a copy of any survey or questionnaire must be attached.

Student Researcher(s):

Title of Project: The Effects of Internet-Based Technology on Human Memory

I am asking for your voluntary participation in my science fair project. Please read the following information about the project. If you would like to participate, please sign in the appropriate area below.

Purpose of the project:
To determine how the ability to search information on the Internet with technology like phones and laptops affects human memory

If you participate, you will be asked to:
complete one or more memory tests, which will determine your capacity to remember different types of information under different conditions (with/without expectation of information being saved, whether or not the information will be stored in a folder, etc.)

Time required for participation:
20-30 minutes

Potential Risks of Study:
No foreseeable risks involved in study

Benefits:
Participants can be provided with a self-assessment on their own capacities to remember different types of information and gain insight into current research within cognitive psychology/neuroscience.

How confidentiality will be maintained:
A Google Form will be used to record the results of the participants, which can only be accessed by myself, the Adult Sponsor, and the Qualified Scientist, and will not be shown to any participants. However, upon request, participants will be able to view their own results.

If you have any questions about this study, feel free to contact:

Adult Sponsor/QS/DS: Phone/email:

Voluntary Participation:
Participation in this study is completely voluntary. If you decide not to participate there will not be any negative consequences. Please be aware that if you decide to participate, you may stop participating at any time and you may decide not to answer any specific question.

By signing this form I am attesting that I have read and understand the information above and I freely give my consent/assent to participate or permission for my child to participate.

Adult Informed Consent or Minor Assent Date Reviewed & Signed:

Research Participant Printed Name: Signature:

Parental/Guardian Permission (if applicable) Date Reviewed & Signed:

Parent/Guardian Printed Name: Signature:

Page 36 *International Rules: Guidelines for Science and Engineering Fairs 2015–2016, student.societyforscience.org/intel-isef*

Prior to taking the memory tests, all participants completed this form to ensure their consent to participate in this study and their understanding of the aims of this study. A digital copy of the form is shown above.

Appendix B

Phase 1 Google Form and Statements

Appendix B.1: Section 1 of the Google Form

The Effects of Internet-Based Technology on Human Memory - Phase 1

Give yourself exactly 10 minutes to read the trivia statements that can be found in this document:
https://docs.google.com/document/d/1SWoNFTaut9YOGBkgdmRdQ7KzlMowEd5BCEc_dUOTZk4/edit
If you are not able to open the document, a screenshot of this document is attached below.

The statements under the "Will Be Saved" column will be stored separately and accessible for future use, and the statements under the "Will Be Erased" column will not be accessible after this reading period. The document with all of the "Saved" statements will be given to you at the end of this form.

* Required

Once your 10-minute reading period is over, continue to the next section of this form. You CANNOT go back to this section after clicking NEXT.

All Statements

Will Be Erased	Will Be Saved
• There are roughly 70 ingredients in the McRib<br>• The pledge of allegiance was written as part of a plan to sell flags to schools.<br>• Alaska is so big you could fit 75 New Jerseys in it.<br>• Apple Pie isn't actually American at all.<br>• The largest known living organism is an aspen grove.<br>• The odds of getting a royal flush are exactly 1 in 649,740.<br>• Four times more people speak English as a second language than as a native one.<br>• A baby puffin is called a "puffling."<br>• Only one NFL team has a plant for a logo.<br>• You can major in wine at Cornell University.<br>• High heel shoes were originally created for men.<br>• The Caspian Sea is the largest enclosed inland body of water on Earth.<br>• Geologic activity around the Ring of Fire is responsible for roughly 90% of all earthquakes worldwide.<br>• The heart of a shrimp is located in its head.<br>• Earth's highest point on land (Mt. Everest), and lowest (Dead Sea), are both found in Asia.	• You can hear a blue whale's heartbeat from more than 2 miles away.<br>• The most shoplifted item in the U.S. is candy.<br>• Some cats are allergic to humans.<br>• The light emitted by 200,000 galaxies makes our universe a shade of beige<br>• The hashtag symbol is technically called an octothorpe.<br>• The unicorn is the national animal of Scotland.<br>• If you drive south from Detroit, you'll hit Canada.<br>• Coca-Cola was the first soft drink in space.<br>• The speed of a computer mouse is measured in "Mickeys."<br>• Pumpkins, squash, and gourds are all technically the same species.<br>• Up to 12 million Dum Dums are made every single day.<br>• Bubble Wrap was originally intended to be used as 3D wallpaper.<br>• At just 135 words, George Washington's second inaugural address remains the shortest ever delivered.<br>• A single cow can make roughly 200,000 glasses of milk in a lifetime.<br>• In 1991, Wayne Allwine, the voice of Mickey Mouse, married Russi Taylor—the voice of Minnie.

Appendix B.2: Section 2 of the Google Form (Memory Questions)

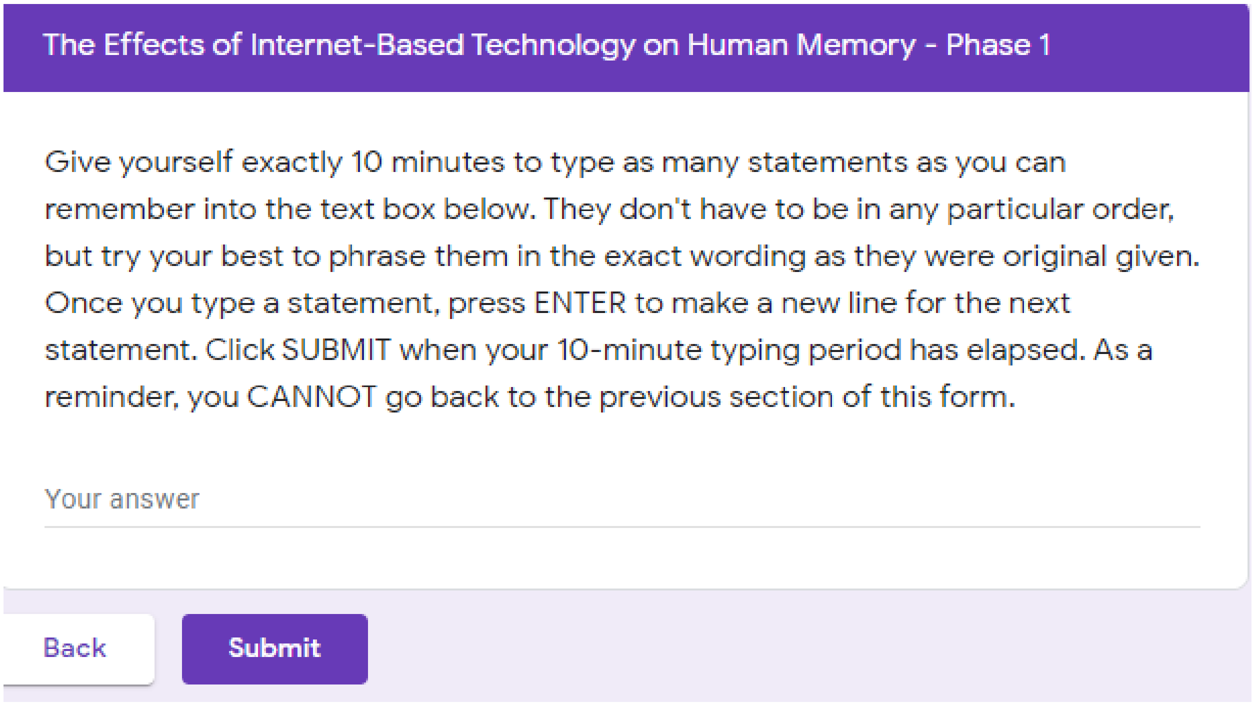
The Effects of Internet-Based Technology on Human Memory - Phase 1

Give yourself exactly 10 minutes to type as many statements as you can remember into the text box below. They don't have to be in any particular order, but try your best to phrase them in the exact wording as they were original given. Once you type a statement, press ENTER to make a new line for the next statement. Click SUBMIT when your 10-minute typing period has elapsed. As a reminder, you CANNOT go back to the previous section of this form.

Your answer

Back

Submit

Participants took this memory test after they were randomly assigned to one of the two groups (explicit memory instructions given or no explicit memory instructions given).

Appendix B.3: “Saved” Statements

- You can hear a blue whale's heartbeat from more than 2 miles away.
- The most shoplifted item in the U.S. is candy.
- Some cats are allergic to humans.
- The light emitted by 200,000 galaxies makes our universe a shade of beige
- The hashtag symbol is technically called an octothorpe.
- The unicorn is the national animal of Scotland.
- If you drive south from Detroit, you'll hit Canada.
- Coca-Cola was the first soft drink in space.
- The speed of a computer mouse is measured in "Mickeys."
- Pumpkins, squash, and gourds are all technically the same species.
- Up to 12 million Dum Dums are made every single day.
- Bubble Wrap was originally intended to be used as 3D wallpaper.
- At just 135 words, George Washington’s second inaugural address remains the shortest ever delivered.
- A single cow can make roughly 200,000 glasses of milk in a lifetime.
- In 1991, Wayne Allwine, the voice of Mickey Mouse, married Russi Taylor—the voice of Minnie.

Appendix C

Phase 1 Raw Data (Google Sheets charts divided by presence of explicit memory instructions)

	Participants with No Explicit Memory Instructions	
	Erased Statements Recalled	Saved Statements Recalled
	5	4
	2	0
	0	2
	4	0
	8	4
	1	0
	8	4
	5	4
	2	2
	4	0
AVERAGE	3.9	2
STANDARD DEVIATION	2.726414006	1.885618083
STANDARD ERRORS	0.8621678104	0.596284794
AVERAGE PROPORTION (out of 15)	0.26	0.1333333333
STANDARD DEVIATION	0.1817609337	0.1257078722
STANDARD ERRORS	0.05747785403	0.0397523196

	Participants with Explicit Memory Instructions	
	Erased Statements Recalled	Saved Statements Recalled
	2	2
	3	1
	5	1
	7	3
	1	4
	4	1
	1	4
	3	2
	7	3
	3	1
AVERAGE	3.6	2.2
STANDARD DEVIATION	2.170509413	1.229272594
STANDARD ERRORS	0.6863753427	0.3887301263
AVERAGE PROPORTION (out of 15)	0.24	0.1466666667
STANDARD DEVIATION	0.1447006275	0.08195150629
STANDARD ERRORS	0.04575835618	0.02591534175

Appendix D

Phase 2 Google Form and Statements

Appendix D.1: Section 1 of the Google Form

The Effects of Internet-Based Technology on Human Memory - Phase 2

Give yourself exactly 10 minutes to read the trivia statements that can be found in this document:
https://docs.google.com/document/d/1GTmVV0qMjdA-5LPXmsIm5VF0zEWRusN_gX1AUuOgC5M/edit?usp=sharing
If you are not able to open the document, a screenshot of this document is attached below.

25 statements will be saved in 4 different folders, and the other 5 statements will be saved in no specific folder. A screenshot of how the statements will be saved is attached below.

All 30 statements and their folder locations will be saved and be accessible for future use after this reading period. The folders and the statements will also be given to you at the end of this form.

Please enter your name and age below. They will remain confidential and will only be used for data analysis purposes.

When your 10-minute reading period is over, continue to the next section of this form. You CANNOT go back to this section once you continue to the next section.

All Statements and Folder Locations

Folder	Statements
Information	• The little BIC pen logo guy's name is BIC Boy.<br>• Dr. Seuss wrote *Green Eggs and Ham* with less than 50 words.<br>• About one in every 4 million lobsters is born with a rare genetic defect that turns it blue.<br>• For one day in 1998, Topeka, Kansas, renamed itself "ToPikachu" to mark Pokemon's U.S. debut.<br>• Only female mosquitoes will bite you.<br>• The space shuttle Columbia disintegrated during reentry over Texas in Feb. 2003
Points	• A bolt of lightning contains enough energy to toast 100,000 slices of bread.<br>• On Good Friday in 1930, the BBC reported, "There is no news." Instead, they played piano music.<br>• Jacuzzi is a brand name.<br>• The only number whose letters are in alphabetical order is 40 (f-o-r-t-y).<br>• Fredric Baur invented the Pringles can.<br>• The duffel bag gets its name from the town of Duffel, Belgium.
Figures	• Reno is farther west than Los Angeles.<br>• Toyota announced that the official plural of Prius was Prii.<br>• The Vatican Bank is the world's only bank that allows ATM users to perform transactions in Latin.<br>• During a 2004 episode of *Sesame Street*, Cookie Monster said that before he started eating cookies, his name was Sid.<br>• A snail can sleep for three years.<br>• In Japan, letting a sumo wrestler make your baby cry is considered good luck.
Facts	• Alaska is the only state that can be typed on one row of keys.<br>• At the 2010 Grammy Awards, Taylor Swift won more Grammys (4) than Elvis did his entire career (3).<br>• Horses are incapable of vomiting.<br>• Hallmark now sells a line of "encouragement" cards you can send to people who've lost their job.<br>• Male students at Brigham Young University need a doctor's note to grow a beard.<br>• The 3 Musketeers bar was originally split into three pieces with three different flavors: vanilla, chocolate and strawberry.
No Specific Folder	• That thing you use to dot your lowercase "i" is called a tittle.<br>• Lyme disease is named after the town of Lyme, Connecticut, where several cases were identified in 1975.<br>• In 1986, Apple launched a clothing line.<br>• Elmo is the only non-human to testify in front of Congress.<br>• An ostrich's eye is bigger than its brain.<br>• Before Stephen Hillenburg created Spongebob Squarepants, he taught marine biology.

Folder Locations

- Points
- Information
- Figures
- Facts
- Phase 2 - Statements Saved in No Specific Folder

Each of these folders contains its respective statements that were accessible to the participants after they completed the memory questions (see **Appendix D.2**).

Appendix D.2: Section 2 of the Google Form (Memory Questions)

The Effects of Internet-Based Technology on Human Memory - Phase 2

Give yourself exactly 10 minutes to answer the following questions IN ORDER. Try your best to type the statements them in the exact wording as they were original given. For the folder location questions, capitalize only the first word of the folder names. Click SUBMIT when your 10-minute answering period has elapsed. As a reminder, you CANNOT go back to the previous section of this form.

Enter the statement about lightning to the best of your ability.

Your answer

In which folder was the statement about lightning saved?

Your answer

In which folder was the statement about the Cookie Monster saved?

Your answer

Enter the statement about the Cookie Monster to the best of your ability.

Your answer

Only the questions on two of the tested statements are shown here. The other questions testing the information on the other eight randomly selected statements follow a similar format.

Appendix D.3: Statements That Were Randomly Selected to be Tested

Question Order: Statement, then folder   Folder, then statement

**Folder**	**Statements**
**Information**	• The little BIC pen logo guy's name is BIC Boy.<br>• Dr. Seuss wrote *Green Eggs and Ham* with less than 50 words.<br>• About one in every 4 million lobsters is born with a rare genetic defect that turns it blue.<br>• **For one day in 1998, Topeka, Kansas, renamed itself "ToPikachu" to mark Pokemon's U.S. debut.**<br>• Only female mosquitoes will bite you.<br>• **The space shuttle Columbia disintegrated during reentry over Texas in Feb. 2003**
**Points**	• A bolt of lightning contains enough energy to toast 100,000 slices of bread.<br>• On Good Friday in 1930, the BBC reported, "There is no news." Instead, they played piano music.<br>• Jacuzzi is a brand name.<br>• The only number whose letters are in alphabetical order is 40 (f-o-r-t-y).<br>• Fredric Baur invented the Pringles can.<br>• The duffel bag gets its name from the town of Duffel, Belgium.
**Figures**	• Reno is farther west than Los Angeles.<br>• Toyota announced that the official plural of Prius was Prii.<br>• The Vatican Bank is the world's only bank that allows ATM users to perform transactions in Latin.<br>• During a 2004 episode of *Sesame Street*, Cookie Monster said that his name was Sid.<br>• A snail can sleep for three years.<br>• In Japan, letting a sumo wrestler make your baby cry is considered good luck.
**Facts**	• Alaska is the only state that can be typed on one row of keys.<br>• At the 2010 Grammy Awards, Taylor Swift won more Grammys (4) than Elvis did his entire career (3).<br>• Horses are incapable of vomiting.<br>• Hallmark now sells a line of "encouragement" cards you can send to people who've lost their job.<br>• Male students at Brigham Young University need a doctor's note to grow a beard.<br>• The 3 Musketeers bar was originally split into three pieces with three different flavors: vanilla, chocolate and strawberry.
**No Specific Folder**	• That thing you use to dot your lowercase "i" is called a tittle.<br>• Lyme disease is named after the town of Lyme, Connecticut, where several cases were identified in 1975.<br>• In 1986, Apple launched a clothing line.<br>• Elmo is the only non-human to testify in front of Congress.<br>• An ostrich's eye is bigger than its brain.<br>• **Before Stephen Hillenburg created Spongebob Squarepants, he taught marine biology.**

The ten statements that were randomly selected to be tested are colored in either red (PSQ) or green (PFLQ).

Appendix E

Phase 2 Raw Data (Google Sheets charts divided by type of information recalled and order of recall)

Number (#), Question (?)	# of Statements for which both Folder/Statement were Recalled		# of Statements for which only Folder was Recalled		# of Statements for which only Statement was Recalled		# of Statements for which Nothing was Recalled	
	Statement ?, then Folder ?	Folder ?, then Statement ?	Statement ?, then Folder ?	Folder ?, then Statement ?	Statement ?, then Folder ?	Folder ?, then Statement ?	Statement ?, then Folder ?	Folder ?, then Statement ?
	0	0	1	1	1	1	3	3
	1	1	0	2	1	0	3	2
	0	0	0	0	2	2	3	3
	0	1	1	0	2	0	2	4
	2	1	1	1	2	1	0	2
	1	0	0	3	1	0	3	2
	0	0	4	5	0	0	1	0
	2	0	0	1	1	1	2	3
	1	0	0	1	4	0	0	4
	2	0	2	1	1	1	0	3
	0	0	1	3	1	0	2	3
	0	1	1	0	2	0	2	4
	0	0	1	3	1	0	2	3
	0	0	1	1	1	1	3	3
	1	0	0	3	1	0	3	2
	0	0	0	0	2	2	3	3
	1	0	0	1	4	0	0	4
	1	1	0	2	1	0	3	2
	0	0	2	5	0	0	3	0
	2	1	1	0	2	1	0	3
	2	0	2	1	1	1	0	3
	2	0	0	2	1	1	2	2
AVERAGE	0.8181818182	0.2727272727	0.8181818182	1.636363636	1.454545455	0.5454545455	1.818181818	2.636363636
STANDARD DEVIATION	0.8528028654	0.4558423058	1.006472559	1.497472618	1.010764573	0.6709817063	1.258735709	1.09307145
STANDARD ERROR	0.1818181818	0.09718590615	0.2145806707	0.3192622349	0.215495731	0.1430537804	0.2683633548	0.2330436162
AVERAGE PROPORTION (out of 5)	0.1636363636	0.05454545455	0.1636363636	0.3272727273	0.2909090909	0.1090909091	0.3636363636	0.5272727273
STANDARD DEVIATION	0.1705605731	0.09116846117	0.2012945119	0.2994945237	0.2021529146	0.1341963413	0.2517471417	0.21861429
STANDARD ERROR	0.03636363636	0.01943718123	0.04291613413	0.06385244699	0.0430991462	0.02861075609	0.05367267096	0.04660872323

Appendix F

Participant Flow Chart Diagrams of Procedural Steps of Phases 1 and 2

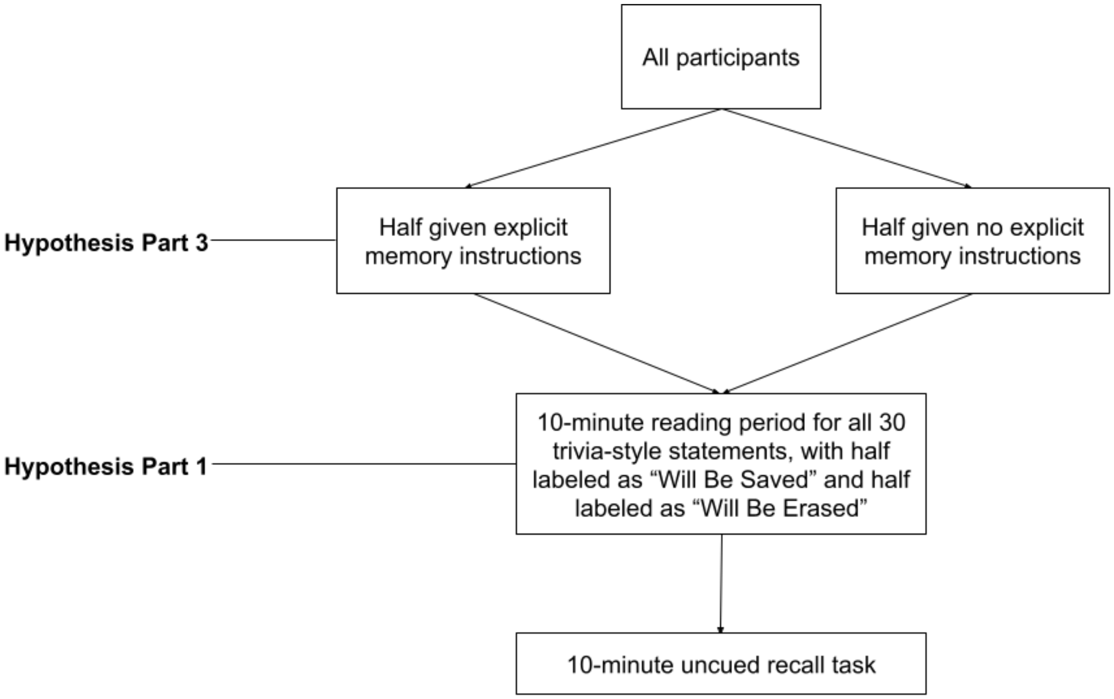

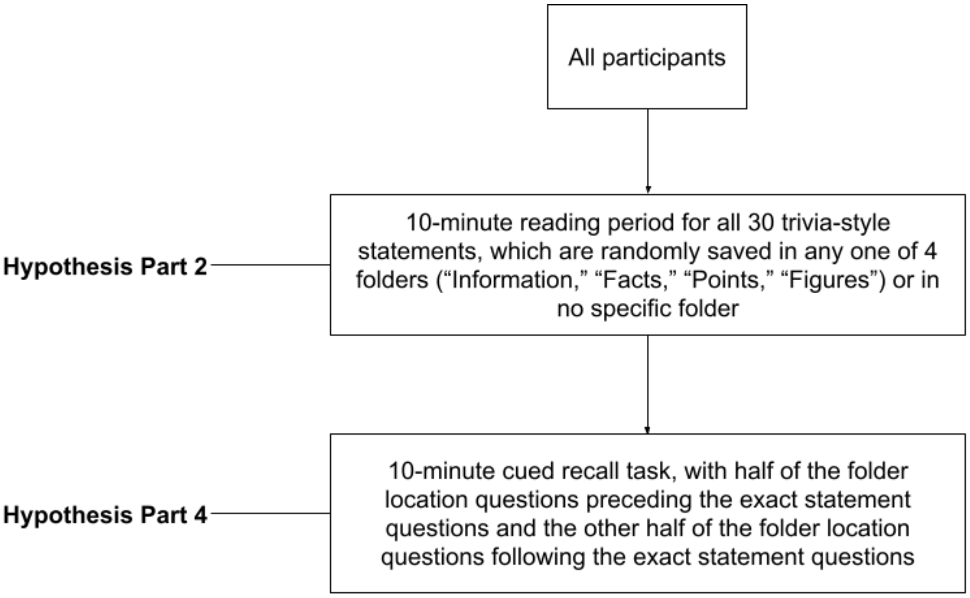

The top participant flow chart diagram provides a visual depiction of the procedural steps of Phase 1, and the bottom participant flow chart diagram depicts those of Phase 2.
